# Analysis of Ammonia Toxicity in Landfill Leachates

**DOI:** 10.5402/2011/954626

**Published:** 2011-09-28

**Authors:** Takuya Osada, Keisuke Nemoto, Hiroki Nakanishi, Ayumi Hatano, Ryo Shoji, Tomohiro Naruoka, Masato Yamada

**Affiliations:** ^1^Department of Chemical Science and Engineering, Tokyo National College of Technology, 1220-2 Kunugida-Machi, Hachioji, Tokyo 193-0997, Japan; ^2^Research Center for Material Cycles and Waste Management, National Institute for Environmental Studies, Onogawa 16-2, Tsukuba, Ibaraki 305-8506, Japan

## Abstract

Toxicity identification evaluation (TIE) phase I manipulations and toxicity test with *D. magna* were conducted on leachates from an industrial waste landfill site in Japan. Physicochemical analysis detected heavy metals at concentrations insufficient to account for the observed acute toxicity. The graduated pH and aeration manipulations identified the prominent toxicity of ammonia. Based on joint toxicity with additive effects of unionized ammonia and ammonium ions, the unionized ammonia toxicity (LC_50,NH_3_(aq)_) was calculated as 3.3 ppm, and the toxicity of ammonium ions (LC_50,NH_4_^+^_) was calculated as 222 ppm. Then, the contribution of ammonia toxicity in the landfill leachate toxicity was calculated as 58.7 vol% of the total toxicity in the landfill leachate. Other specific toxicants masked by ammonia's toxicity were detected. Contribution rate of the toxicants other than by ammonia was 41.3 vol% of the total toxicity of the landfill leachate.

## 1. Introduction

Landfill leachates are highly polluted effluents [1]. The leachate has to be treated in a downstream waste water treatment plant, and it is necessary to identify toxicity causative chemicals for the effective treatment. Accordingly, methods were developed to assist in identifying effluent toxicants, and these methods are well known as a toxicity identification evaluation, TIE. TIE methods have been proven to be effective tools for characterizing and identifying toxicants in samples of effluents and other complex mixtures [2]. Two types of TIE, treatability-based TIE and chemical-specific TIE, have been studied so far. Treatability-based TIE is a general approach in TIE to determine the effective water treatments and (if possible) thereby speculate the toxicity-controlling chemical(s). Chemical-specific TIE was developed to evaluate the toxicity-contributed chemicals more simply. The approach is to compare chemical analysis data with concentration of chemicals to express toxicity (e.g., LC_50_, EC_50_ (the lethal or effective concentration for 50% of test organisms), etc.). However, these TIEs have some problems. First, in the treatability-based TIE, when the chemical which highly contributed to the toxicity was included in the sample, the toxicity of other chemical cannot be detected by the TIE based on a single toxicity test. Second, in the chemical-specific TIE, when concentration of many chemicals were higher than LC_50_ or EC_50_, toxicity causative chemicals for effective treatment cannot be identified. Third, environmental factor (e.g., pH) cannot be considered. Ionized and un-ionized forms of the compounds such as heavy metals have different toxicity to the organisms. The ionizable compounds are commonly found in landfill leachate including ammonia and some organic compounds as well as heavy metals. In addition, pH affects metal toxicity through changes in solubility and speciation [3, 4]. Contribution rate is one of the quantitative approaches to solve the above-mentioned problems. The contributions of each component were calculated as quotients of concentration, though contribution rate has not been used for identification of toxicity-causative chemicals. Therefore, contribution rate approach for identification of toxicity causative chemicals can consider the effect of pH in landfill leachate toxicity for more effective treatment.

This manuscript describes toxicity testing and TIE studies conducted on industrial waste landfill leachate. Through this case study, we describe the problems of TIE testing used to characterize, identify, and confirm ammonia as the cause of acute toxicity to the *Daphnia magna*. Ammonia toxicity considering pH in the landfill leachate was analyzed. The objectives of this study are to perform contribution rate approach for identification of toxicity causative chemicals considering the effect of ammonia toxicity changing by pH, and to detect toxicity causative chemicals other than ammonia with the contribution rate approach.

## 2. Materials and Methods

### 2.1. Effluent Sampling and Chemical Analysis of Landfill Leachate

Two effluents were collected from the same landfill site in Japan in 2008 and 2009. The landfill site for final disposal of industrial wastes has already been closed since the 1990s. Metal concentrations were determined with an ICP-AES (SII SPS7800) in landfill leachate in 2008, and an ICP-MS (Agilent 7500s, YOKOGAWA) in landfill leachate in 2009. The ammonium ions and other ionic chemical parameters (F^−^, Cl^−^, NO_2_^−^, Br^−^, SO_4_^2−^, Na^+^, K^+^, Mg^2+^, and Ca^2+^) concentrations were determined by using an ionic chromatograph (TOSOH IC-2001). Total organic carbon concentration (TOC) was also analyzed by using a TOC analyzer (TOC-5000A, Shimadzu). Before these physico-chemical analysis, samples were filtered with a 0.45 *μ*m paper filter. In ICP-AES, samples were acidified with twentyfold diluted 65 wt% nitric acid.

### 2.2. Acute TIE Manipulations

Acute TIE studies began with a full phase I toxicity characterization as described by USEPA [2]. This procedure involves some different tests, which evaluate the effect of physical/chemical manipulations on effluent toxicity. Comparing the toxicity of manipulated samples with that of unmanipulated effluent provides information on the physical/chemical properties of the specific toxicant(s). Baseline effluent toxicity test was conducted at effluent dilutions of 100 vol%, 50 vol%, 25 vol%, 12.5 vol% and 6.25 vol%. pH adjustment was used throughout phase I to provide more information on nature of the toxicants. Changes in pH can affect the solubility, polarity, volatility stability, and speciation of a compound, thereby affecting its bioavailability as well as its toxicity. One molar of NaOH or 1.0 M HCl was added dropwise to the samples to control the pH near 11 or 3. The aeration test is designed to determine how much effluent toxicity can be attributed to volatile, sublatable, or oxidizable compounds. The pH of the acidic and basic effluent and dilution water aliquots should be checked every 5 min during the first 30 min of aeration and every 10 min thereafter. If the pH 3 or pH 11 solution drifts more than 0.2 pH units, it must be readjusted back to the nominal value. The graduated test pH test was also modified by performing the test at pH 6.5, 7.5, and 8.5. Since ammonia toxicity significantly varies over this range of pH values, the relative difference in toxicity could still be examined. EDTA is a strong chelating agent, and its addition to water solution produces relatively nontoxic complexes with many metals. Oxidant reduction test was designed to determine to what extent constitutes reduced by the addition of sodium thiosulfate are responsible for effluent toxicity. Concentration of sodium thiosulfate equal to and lower than the thiosulfate LC_50_ for test species being used are added to several containers with effluent at the 100% concentration. In addition to the phase I manipulations described by USEPA, other manipulations were conducted to further characterize the effluent toxicant(s). Filtration manipulations tests were conducted (MF  =  microfiltration; pore diameter 0.45 *μ*m, UF  =  ultrafiltration; membrane area 0.40 m^2^ pressure 0.40 MPa (RF002040, ADVANTEC MFS, INC. Tokyo, Japan), RO = reverse osmosis; membrane area 0.40 m^2^ pressure 0.40 MPa (RF000670, ADVANTEC MFS, INC. Tokyo, Japan)).

### 2.3. Ammonia Toxicity Testing and Analysis

In aqueous solution, un-ionized ammonia (NH_3 _(aq)) exists in equilibrium with the ammonium ion (NH_4_^+^) according to the dissociation equation:

(1) $$\;{{\text{NH}}_{4}}^{+}+{\text{H}}_{2}\text{O}\overset{\text{Ka}}{⟷}\text{N}{\text{H}}_{3}\left(\text{ap}\right)+{{\text{H}}_{3}\text{O}}^{+}.$$

Total ammonia concentration is the sum of un-ionized and ionized ammonia. The toxic effect of total ammonia increases with increasing pH, indicating that the un-ionized ammonia is the main toxic form. Un-ionized ammonia concentration was calculated by using ion speciation analysis software MINEQL+ (Environmental Research Software) and ammonium ion concentration measured in this study. To specify the ammonia toxicity, samples with pure ammonium chloride were adjusted in terms of pH at 7.0 and then the toxicity was tested with *D. magna*.

*D. magna* acute test was performed according to OECD guideline 202, at adjustment of pH using diluted NaOH or HCl solution. Test organisms originated from a healthy* D. magna* clone which has been cultured in the laboratory under standardized conditions in the ISO test solution (2.00 × 10^−3^ M CaCl_2_, 5.00 × 10^−4^ M MgSO_4_·7H_2_O, 7.70 × 10^−4^ M NaHCO_3_, and 7.71 × 10^−5^ M KCl). Acute toxicity was assessed by noting the effects of the test compounds on the effective concentration of *D. magna*. The tests were conducted at a constant temperature of 20 ± 1°C. *D. magna* were exposed to each landfill leachates, ammonia, and bisphenol A for 48 hour of exposure duration. The number of mortality *D. magna* was recorded. The acute toxicity endpoint was determined as the LC_50_ of a chemical that causes 50% of reduction of *D. magna* survival. The toxicity of the target chemicals have been evaluated as the influence of matrix effects for the determination of LC_50_ values. The biological response is correlated to the toxicant concentration. The logistic dose response relationship is described as follows [5]:

(2) $$R=\frac{100}{1+{\left(x/x_{50}\right)}^{\beta }},$$

where *R* = biological response as percentage of the mortality rate of *D. magna*, *x* was concentration of toxicant, and *x*_50_  was the concentration of poison that resulting 50% reduction of survival in *D. magna*, *β* is the shape parameter of the dose response curve.

A simple model to describe the toxicity of a protolyzing substance is based on its dissociation and the addition of each form in the dissociation [6]:

(3) $$\begin{array}{cc} & \frac{1}{\text{L}{\text{C}}_{50,\text{ tot}}}=\frac{1}{\text{L}{\text{C}}_{50,{\text{N}{\text{H}}_{\text{4}}}^{\text{+}}}}-\left(\frac{1}{\text{L}{\text{C}}_{50,{{\text{NH}}_{4}}^{+}}}-\frac{1}{\text{L}{\text{C}}_{50,{\text{N}{\text{H}}_{4}}^{+}(\text{aq})}}\right)\\ & \;\;\;\;\times \frac{\text{Ka}}{\text{Ka}+{\text{C}}_{H^{+}}}. \end{array}$$

  LC_50,tot_, LC_50,NH_3_(aq)_, and LC_50,NH_4_^+^_ were 50% effective concentration expressed as total ammonia, un-ionized ammonia and ammonium ion, respectively, Ka was stability constant for NH_3_ and NH_4_^+^, C_*H*^+^_ was concentration of hydrogen-ion. From a linear fit with data of the toxic effect as 1/LC_50,tot_ against dissociation expressed as dissociation of ammonium, the toxicity of the un-ionized ammonia is obtained from the intercept, and the toxicity of the ammonium ion is obtained from the slope and the intercept.

### 2.4. Contribution Rate Calculation

The toxic contribution of the specific toxicants in the landfill leachate was derived by calculations of contribution rate at the LC_50_ concentration of the leachate (% v/v). First, the concentrations of toxicity causative chemicals were measured in the landfill leachate (measured), then the concentrations of the the toxicity causative chemicals in the LC_50_ mixture (mixture) were calculated. Then, for each toxicity causative chemicals, the mixture was compared with the specific LC_50_ of each toxicity causative chemicals:

(4) $$\begin{array}{cc} & \frac{\text{Landfill}\;\text{leachate}\;{\text{LC}}_{50,{{\text{NH}}_{4}}^{+}\;\text{or}\;{\text{NH}}_{3}(\text{aq})}}{{\text{LC}}_{50,{{\text{NH}}_{4}}^{+}\;\text{or}\;{\text{NH}}_{3}(\text{aq})}}\times 100\\ & \;=\text{Contribution on rate of}\;{{\text{NH}}_{4}}^{+}\;\text{or}\;{\text{NH}}_{3}\left(\text{aq}\right)\;\left[\text{vol}\%\right]. \end{array}$$

## 3. Results and Discussion

### 3.1. Landfill Leachate Characterization and Chemical-Specific TIE Approach

Table 1 shows physico-chemical analysis of industrial waste landfill leachate and LC_50_ values of each chemical components for *D. magna*. Landfill leachates showed high ammonia concentration and elements of salinity concentration, such as potassium and sodium. Additionally, heavy metal concentrations in both landfill leachate samples were lower than each LC_50_.

Physico-chemical analysis data with LC_50_ value in each components were compared for chemical-specific TIE approach, then the results showed that heavy metals were not toxicity causative chemicals. Unfortunately, many kinds of toxicity causative chemicals were derived by the chemical-specific TIE approach. Another trial on the chemical-specific TIE performed by Shoji et al. provided precious information on the toxicity-controlling chemicals in landfill leachates [7]. Twenty-five landfill leachate samples were examined by a toxicity test and chemical analyses [7]. By comparing two important parameters describing dose-response relationship (the EC_50_ value and the slope) between landfill leachate sample and 255 kinds of chemicals, possible candidates of toxicity-controlling chemical were listed as bisphenol A and other phenols. Subsequently performed chemical analyses successfully showed the presence of such chemicals, and the concentration of these chemicals partly explained the observed toxicity. In order to take an effective countermeasure for the waste landfill leachate, both chemical analyses and toxicity tests can provide important information to find the targeted and cost-effective water treatment.

### 3.2. Treatability-Based TIE Approach

**Figure 1:**
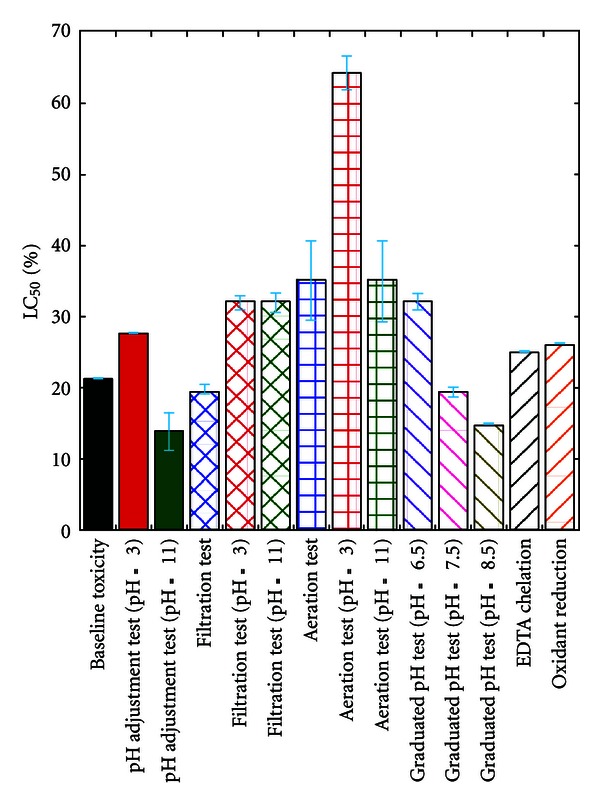
Results of acute phase I characterization testing and supplemental characterization studies* Daphnia magna*.

Figure 1 shows results of acute phase I characterization testing with a *D. magna* immobilization test. The baseline LC_50_ for *D. magna* was approximately 20 vol% of the landfill leachate. Toxicity of solid phase extraction-treated leachate and EDTA-addition leachate were not reduced compared to that of untreated leachate. Aeration manipulation tests showed a clear decrease in toxicity. The acutely toxic landfill leachate was submitted for chemical analysis shown in Figure 1; ammonia was detected in the sample, and the concentration was 361 ppm considerably in excess of concentrations to be toxic to *D. magna*. Toxicity of manipulated sample relevant to pH-change was reduced relative to untreated leachate, reinforcing our suspicion that the toxicant was ammonia. In another case of treatability-based TIE, Stronkhorst et al. indicated that tests using graduated pH manipulations showed significant increase in toxicity from low pH to high pH in a sediment samples in silty marine harbor dominated by ammonia or sulfide [8].

### 3.3. Analysis of Ammonia Toxicity

**Figure 2:**
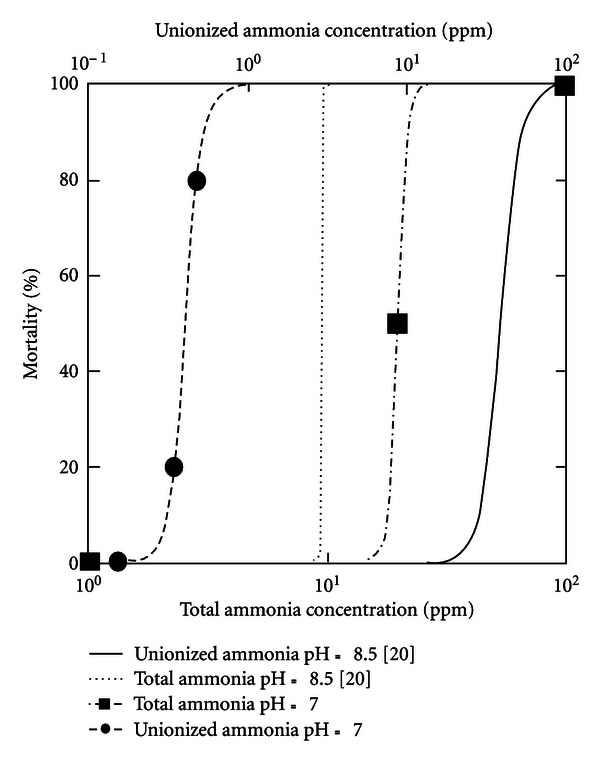
Effect of ammonia on the mortality rate of Daphnia magna at pH 7.0 and 8.5 [20]. Concentration expressed as total ammonia or as un-ionized ammonia calculated by MINEQL+.

In terms of the influence of pH conditions on the proportion of ammonium ions to total toxicity of ammonia, it was necessary to perform the toxicity tests with *D. magna* at defined pH in order to elucidate the toxicity of ammonia. Examples of concentration/response curves obtained in terms of ammonium chloride at pH 7.0 and 8.0 are shown in Figure 2. Un-ionized ammonia constituted the major source of toxicity in these investigations, even though volume of un-ionized ammonia comprised a small fraction of total ammonia, in term of a mixture of two toxic components at different proportions assuming additive effects of the two forms. 

**Figure 3:**
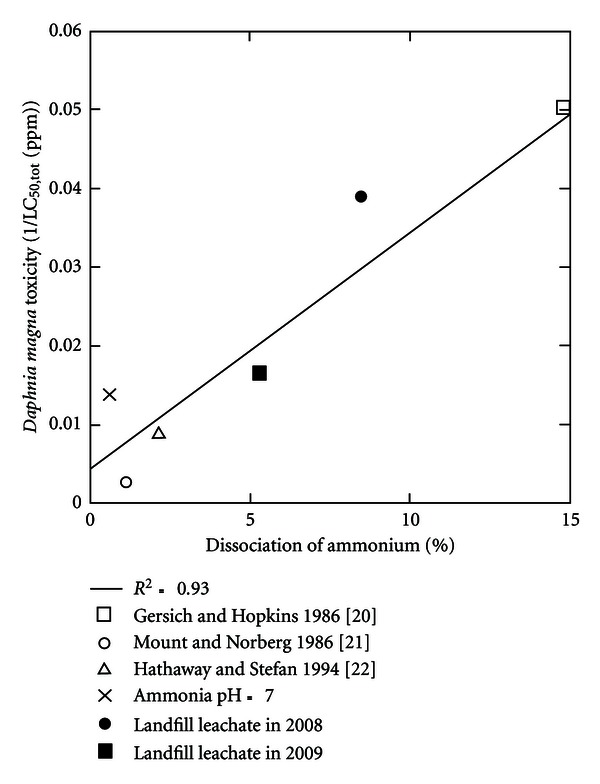
Ammonia toxicity in tests with *Daphnia magna* at different pH related to percentage of dissociation of ammonia [20–22].

The specific toxicity of un-ionized ammonia and ammonium ion were calculated from a plot of the inverse of LC_50_ versus the degree of dissociation (Ka/Ka + C_*H*^+^_) according to (3) as shown in Figure 3. The toxicity of ammonia and ammonium ion mixture increased upon increasing pH. From a linear fit with data of the toxic effect as 1/LC_50,tot_ against dissociation expressed as dissociation of ammonium, the correlation coefficient (R^2^) between observed plots and the linear function expressed as (1) was 0.93. The toxicity of the un-ionized ammonia is obtained from the intercept, and the toxicity of the ammonium ions from the slope and the intercept. The un-ionised ammonia toxicity (LC_50,NH_3_(aq)_) was calculated as 3.3 ppm and the toxicity of ammonium ions (LC_50,NH_4_^+^_) was calculated as 222 ppm, that is, almost a factor 50 difference between (LC_50,NH_3_(aq)_) and (LC_50,NH_4_^+^_). Joint toxicity with these components have been observed by an algae *Nephroselmis pyriformis*, within almost a factor 100 difference between (LC_50,NH_3_(aq)_) and (LC_50,NH_4_^+^_). Ammonium ion is slightly toxic among different species of ammonia, but concentration of ammonium ion was much more larger than that of un-ionized ammonia around pH 7. The contribution of ammonium ions in the total ammonia toxicity was therefore not negligible. According to some previous studies on mixture toxicity of chemicals [9], the multiple toxicity caused by two or more chemicals can be classified into three types, additive, synergistic, and antagonistic effects. The multiple toxicity of ammonia and ammonium ion can be assumed as an additive effect [10]. As discussed above, various chemicals other than ammonia are contained in landfill leachate. Information on multiple toxicity are too limited to discuss more on interaction among other heavy metals on organic chemicals such as bisphenol A.

### 3.4. TIE Based on Contribution Rate Approach

The contribution of each component in the landfill leachate toxicity were calculated according to (3), (4), and specific ammonia toxicity, and the results are presented in Table 2. Table 2 also indicates that ammonia is a main toxic constituent in the landfill leachate. Treatability-based TIE led the fact that toxicity causative chemical is only ammonia. In landfill leachate in 2008, contribution rate of ammonia toxicity was calculated as 58.7 vol%. Contribution of other toxicity causative chemicals should be masked by the ammonia toxicity. The toxicity contribution of chemicals other than ammonia was calculated as 41.3 vol%. In landfill leachate in 2009, the sum of contribution rate and the lack of correlation to concentration does not allow a precise evaluation, because other components may interfere with the toxic action of ammonia. Previous papers suggest that ammonia toxicity to amphipod and fish was strongly dependent on the ionic composition of the medium (e.g., potassium and sodium ion) [11–13].

As a toxicity causative chemical other than ammonia, bisphenol A (BPA) was suggested by chemical-specific TIE based on the concentration shown in Table 2. Table 2 shows the contribution of BPA toxicity in the total toxicity of landfill leachate. Contribution rate of BPA toxicity was calculated as 8.21 vol% in landfill leachate in the year of 2008, 29.0 vol% in landfill leachte in the year of 2009. Although BPA was one of the toxicity causative chemicals in the landfill leachate, BPA toxicity was not determined by the SPE manipulation tests of treatability-based TIE, because toxicity of ammonia was relatively large so that the toxicity of BPA was shadowed. According to the previous studies on the toxicity of BPA, the acute toxicity data showed that BPA was moderately toxic to the invertebrates tests [14]. Shoji et al. tried to find toxicity-controlling chemicals in waste landfill leachate [7] and waste samples such as various sludges [15]. According to their findings, BPA was found as possible candidates for toxicity-controlling chemicals in landfill leachate and parts of waste sludge samples, respectively. In addition, the contribution of ammonia to landfill leachate toxicity was already examined in a previous study. Un-ionized ammonia was a more toxic form of ammonia and seemed to be the major toxicant for most leachates from 16 landfill sites [1].

## 4. Conclusions

A methodology to decide toxicity causative chemicals in landfill leachate based on the toxicity contribution rate and the effect of ammonia toxicity changing by pH was developed in this study. The contribution of ammonia toxicity in the landfill leachate toxicity was calculated as 58.7 vol% of the total toxicity of the landfill leachate. Other toxicity causative chemicals masked by ammonia's toxicity were detected. Contribution rate of the toxicity causative chemicals masked by ammonia toxicity was 41.3 vol% of the total toxicity of the landfill leachate. Bisphenol A was one of the toxicity causative chemicals other than ammonia in this study. TIE based on contribution rate approach developed in this study enables us to detect toxicity causative chemicals masked by high contribution chemicals independently of pH of landfill leachate.

## Figures and Tables

**Table 1:** Chemical analysis of waste landfill leachate and LC50.

Chemical	Landfill leachate (ppm)		LC_50_ of *Daphnia magna *
	2008	2009	(literature values) (ppm)
NO_3_^−^	N.D.	N.D.	3581 [16]
SO_4_^2−^	17.7	2530	2560 [16]
Na^+^	1760	3130	3310 [16]
NH_4_^+^	334	361	25.7
K+	102	380	337 [16]
TOC	147	152	—
Cu	2.25 × 10^−5^	3.48 × 10^−2^	5.73×10^−2^ [17]
Zn	N.D.	4.73 × 10^−2^	3.34×10^−1^ [18]
Ni	N.D.	5.64 × 10^−2^	1.66×10^−1^ [18]
Pb	3.19 × 10^−2^	3.70 × 10^−2^	4.97 [19]
Bisphenol A	16.3	14.8	15.2

pH	8.3 (±0.3)	7.7 (±0.1)	—

N.D. = not detected: detection limits of NO_3_^−^, Zn, and Ni were 0.038 ppm, 0.004 ppm, and 0.006 ppm ([16] Dowden and Benette 1965. [17] Naddy, et al. 2002. [18] Chapman, et al. 1980. [19] Elten-Unal, et al. 1998.)

**Table 2:** Contribution of ammonia and bisphenol A toxicity in the total toxicity of landfill leachate.

Landfill leachate	pH	Daphnia magna, LC50 (ppm)			Contribution rate (vol%)				
		NH_4_^+^	NH_3_ (aq)	Total ammonia	NH_4_^+^	NH_3_(aq)	Total ammonia	Bisphenol A	Bisphenol A and ammonia
2008	8.3 (±0.3)	19.3	1.64	20.9	8.7	50	58.7	8.21	66.9
2009	7.5 (±0.1)	110	2.35	112	49.5	71.1	121	29.0	150
